# Report of Deaths in the City of Buffalo, for the Month of April, 1862

**Published:** 1862-06

**Authors:** 


*Report of Deaths in the City of Buffalo, for the Month of April,* 1862.

Abscess, 1; Accident, 8; Accident by drowning, 6; Apoplexy, Cerebral, 1; Asthma, 2; Bronchitis, 1; Cancer, 1; Consumption, 23; Convulsions, 8; Croup, 3; Delirium Tremens, 5; Dentition, 1; Diarrhœa, 2; Diphtheria, 2; Dropsy, general, 1; Dropsy of the Brain, 6; Dysentery, 1; Empyema, 1; Fever, 2; Fever, Scarlet, 4; Fever, Typhoid, 6; Fever, Typhus, 2; Gangrene, 2; Hemorrhage, 1; Hemorrhage from Lungs, 1; Inflammation of Brain, 3; Inflammation of Brain and Meninges, 2; Inflammation of Larynx, 1; Inflammation of Liver, 1; Inflammation of Lungs 7; Inflammation of Lungs, Typhoid, 1; Inflammation of Peritoneum, 1; Inflammation of Stomach, 1; Intemperance, 1; Kidneys, Bright's Dis; of, 1; Old Age, 3; Otitis, 1; Paralysis, 1; Purpura Hemorrhagica, 1; Scrofula, 1; Tabes Mesenterica, 1: Uuknown, 4; Whooping Cough, 1;—Total 125. Ages, between 1 and 30 days, 1; between 1 and 6 months, 8; between 6 months and 1 year, 10; between 1 and 3 years, 20; between 3 and 5 years, 8; between 5 and 10 years, 7; between 10 and 20 years, 9; between 20 and 30 years, 15; between 30 and 40 years, 14; between 40 and 50 years, 11; between 50 and 60 years, 9; between 60 and 70 years, 6; between 70 and 80 years, 5; over 80, 1; Still-born, 6, Unknown, 1; Total 131.

